# A fractional diffusion random laser

**DOI:** 10.1038/s41598-019-44774-3

**Published:** 2019-06-18

**Authors:** Yuyao Chen, Alfredo Fiorentino, Luca Dal Negro

**Affiliations:** 1 Boston University, Department of Electrical and Computer Engineering and Photonics Center, 8 Saint Mary’s Street, Boston, Massachusetts 02215 USA; 2 Università di Catania, Dipartimento di Fisica e Astronomia “Ettore Majorana”, via S. Sofia, 64, 95123 Catania, Italy; 3 Università di Catania, Scuola Superiore di Catania, Via Valdisavoia, 9, 95123 Catania, Italy; 4 Boston University, Department of Physics, 590 Commonwealth Avenue, Boston, Massachusetts 02215 USA; 5 Boston University, Division of Material Science and Engineering, 15 Saint Mary’s Street, Boston, Massachusetts 02446 USA

**Keywords:** Optical physics, Optics and photonics

## Abstract

The goal of this letter is to introduce the concept of a non-resonant fractional random laser. This is achieved by extending the classical Letokhov model of photon diffusion through disordered gain media to fractional differential operators in space and time. Fractional transport equations effectively describe anomalous photon sub-diffusion phenomena in non-uniform random scattering media with memory and long-range spatial correlation effects. In particular, by analytically solving fractional transport equations in the one-dimensional slab geometry we obtain simple closed-form expressions for the critical amplification volumes required to initiate the laser action in both fractional-order (FO) and distributed-order (DO) space-time fractional reaction-diffusion equations. Our findings demonstrate the benefits of anomalous sub-diffusive photon transport in active media with correlated disorder and stimulate the engineering of novel non-resonant random lasers with significantly reduced footprint and amplification volumes beyond the limitations of uniform disorder and Markovian diffusion processes.

## Introduction

Traditional lasers require a gain medium and the implementation of a positive feedback loop. Typically, positive feedback is achieved by positioning the amplifying optical medium in between two parallel and highly reflective mirrors forming an optical cavity. At each photon round-trip the confined cavity radiation is amplified by the gain medium and gives rise, above a characteristic threshold, to the laser action. However, it was realized already in 1966 by Ambartsumyan *et al*.^1^ that by replacing one cavity mirror with a scattering surface a non-resonant, intensity-based feedback loop could be realized in which the radiation does not retrace its original position after one round-trip. In this case, a large number of spectrally overlapping resonances with a low quality factor *Q* is excited, forming a continuous spectrum in which the only resonant element is the spectral linewidth of the gain medium. The idea of a non-resonant photon feedback was further developed by Letokhov^2^ who in 1968 theoretically considered light amplification in a gain medium of random scatterers within the classical photon diffusion picture valid when the photon mean free path is shorter than the linear dimension of the scattering medium and much longer than the optical wavelength. The resulting *incoherent feedback* random lasers have been experimentally demonstrated in the mid 1980s using *Nd*^3 +^ -doped scattering powders^3^ and in the early 1990s with the discovery of laser paints^4,5^, where the scattering and the amplification medium are separate. In 1998 a new kind of random laser with a phase-sensitive coherent feedback due to light trapping by recurrent scattering events has been experimentally demonstrated in semiconductor materials^6,7^. We note that the distinction between coherent and incoherent feedback mechanisms should not be considered as a fundamental one since interference effects are always present in the multiple scattering regime^8^. In fact, a more fundamental distinction would be the one between lasers that can be modeled using integer or fractional diffusion models and lasers whose description requires more accurate ab-initio methods based on the solutions of full-vector Maxwell’s equations in dielectric systems with randomly varying refractive index^9–14^. Currently, random lasers are investigated in a large number of material systems, and have attracted a significant interest due to their ease of fabrication and robustness combined with a small-size (micron-scale) offering unique characteristics such as low degree of spatial coherence, lack of directionality, bio-compatibility, etc. These are very promising features for a number of engineering applications to bio-sensing, medical diagnostics, on-chip spectroscopy, and optical imaging^15,16^.

In this letter, by focusing exclusively on random lasers with incoherent feedback that are described within the diffusion approximation, we propose to extend the traditional Letokhov model by considering fractional diffusion equations of arbitrary order, including distributed-order (DO) fractional diffusion processes. In recent years, a powerful mathematical framework was introduced in applied sciences and engineering that makes use of kinetic macroscopic (transport) equations with generalized derivatives and integrals of fractional order^17,18^. This approach exploits the mathematics of fractional calculus and provides an efficient analytical description of complex anomalous transport phenomena in situations where long-range memory and spatial correlation effects become important and the traditional (Markovian) approach to uniform random media is simply inadequate. Fractional diffusion equations involve integro-differential operators with non-local power-law kernels that physically account for space-time correlations in the scattering events within non-homogeneous or correlated random environments. Non-local effects in multiple light scattering naturally arise due to the fact that the mean field at a given position inside a complex medium generally depends on the distribution and spatial correlation of the surrounding scattering particles as well. In the case of structurally complex, nonuniform photonic random media and metamaterials with structural correlations the onset of such non-local effects poses fundamental challenges to the traditional numerical analysis techniques, motivating the development of alternative approaches based on macroscopic transport models of fractional order^19^. Our comprehensive analytical and numerical analysis of fractional photon transport regimes in gain media demonstrates the relevance of engineered photon sub-diffusion processes to realize novel lasers and complex aperiodic structures with strongly reduced amplification threshold and footprint.

## The Letokhov Diffusion Model

We begin our discussion by first reviewing the main features of the Letokhov diffusion model of a non-resonant random laser, which provides the necessary background to appreciate our subsequent fractional generalizations. Mathematically, photon diffusion through a uniform disordered random medium with optical gain is described by a reaction-diffusion equation that supports exponentially increasing solutions beyond a critical amplification volume *V*_*cri*_, as first realized by Letokhov in his 1968 seminal paper^2^. The classical Letokhov model of a non-resonant random laser is formulated in terms of the following reaction-diffusion equation obeyed by the optical energy density:

1 $$\frac{\partial W(\overrightarrow{r},t)}{\partial t}=D{\nabla }^{2}W(\overrightarrow{r},t)+\frac{v}{{\ell }_{g}}W(\overrightarrow{r},t)$$

where *D* is the diffusion constant of photons, *v* is the speed of light in the medium, and ${\ell }_{g}$ is its characteristic gain length. Following ref.^2^ in our work we will consider ${\ell }_{g}\gg {\ell }_{t}$, where ${\ell }_{t}$ is the transport mean free path. Furthermore, we have that $D=v{\ell }_{t}/2n$^17^, where *n* is the dimensionality of the problem. In order to more clearly understand the role of fractional operators on the critical volume for light amplification we will focus on one-dimensional (1D) random media. Moreover, it will be convenient to work with scaled variables in order to generalize our treatment to fractional operators. Specifically, we will consider the scaling ${\tau }_{d}=\frac{{\ell }_{t}}{v}$ and ${\tau }_{g}=\frac{{\ell }_{g}}{v}$, which are the characteristic time for the scattering and the amplification time of a photon, respectively. In these transformed variables Eq. (1) by *τ*_*d*_ can be rewritten as:

2 $$\frac{\partial W^{\prime} (x,t^{\prime} )}{\partial t^{\prime} }=D{\tau }_{d}\frac{{\partial }^{2}W^{\prime} (x,t^{\prime} )}{\partial {x}^{2}}+\frac{{\tau }_{d}}{{\tau }_{g}}W^{\prime} (x,t^{\prime} )$$

where we additionally defined the scaled time variable as $t^{\prime} =\frac{t}{{\tau }_{d}}$, and *W*^′^(*x*, *t*′) = *W*(*x*, *t*). We notice that the gain coefficient can now be expressed as $\frac{{\tau }_{d}}{{\tau }_{g}}=\frac{{\ell }_{t}}{{\ell }_{g}}$, which will turn out to be a fundamental parameter in our description of the fractional random laser regimes. The equation above can be solved by the separation of variables method, which yields the separated form as:

3 $$W^{\prime} (x,t^{\prime} )=\sum _{n}\,{a}_{n}{{\rm{\Psi }}}_{n}(x){e}^{-({B}_{n}^{2}D{\tau }_{d}-{\tau }_{d}/{\tau }_{g})t^{\prime} }$$

where Ψ_*n*_(*x*) and *B*_*n*_ are the eigenfunctions and the eigevalues of the following equation:

4 $$\frac{{d}^{2}{{\rm{\Psi }}}_{n}(x)}{d{x}^{2}}+{B}_{n}^{2}{{\rm{\Psi }}}_{n}(x)=0$$

and $W^{\prime} (x,0)=\sum _{n}\,{a}_{n}{{\rm{\Psi }}}_{n}(x)$. The constants *a*_*n*_ and *B*_*n*_ are determined by the choice of the initial-boundary conditions. This solution can be readily generalized to the three-dimensional random media by considering the equation ${\nabla }^{2}{\rm{\Psi }}+{B}_{n}^{2}{\rm{\Psi }}=0$ instead. In that case, the method of separation of variables represents the solution in the form *W*(*x*, *y*, *z*, *t*) = Ψ(*x*)Θ(*y*)Λ(*z*)*f*(*t*′), which reduces to the solution of the 1D problem *W*(*x*, *t*) = Ψ(*x*)*f*(*t*′) in each spatial dimension.

The boundary condition Ψ_*n*_(*x*) = 0 is typically imposed at the extrapolation length *x*_*e*_ beyond the physical border of the scattering medium. Since ${x}_{e} \sim {\ell }_{t}\ll L$ we assume for simplicity Ψ(0) = Ψ(*L*) = 0. Here *L* is the total length of the 1D system. By imposing the boundary conditions, we obtain ${B}_{n}=\frac{n\pi }{L}$, where *n* is a positive integer. It is important to realize that the time-dependent part of the solution of Eq. (3) switches from an exponential decay to an exponential growth at a threshold value determined by:

5 $$-{B}_{1}^{2}D{\tau }_{d}+\frac{{\tau }_{d}}{{\tau }_{g}}\ge 0\Rightarrow -\,{B}_{1}^{2}D+v/{\ell }_{g}\ge 0$$

where *B*_1_ = *π*/*L* is the lowest-order (*n* = 1) eigenvalue. Therefore, a critical volume ${V}_{cri}\approx {L}_{cri}^{3}$ can be defined for the laser action according to:

6 $${V}_{cri}\approx {\pi }^{3}{(D\frac{{\ell }_{g}}{v})}^{\frac{3}{2}}={\pi }^{3}{(\frac{{\ell }_{t}{\ell }_{g}}{2})}^{\frac{3}{2}}$$

In the following sections we will generalize the Letokhov model using fractional operators and obtain analytical expressions for the critical volume in sub-diffusive and super-diffusive transport regimes that can be efficiently modelled by considering fractional reaction-diffusion equations.

## Fractional Random Lasers: Single-Order Sub-Diffusive Regime

The goal of our paper is to study the lasing properties of random systems governed by anomalous diffusion. We will assume ${\ell }_{g}\gg {\ell }_{t}$ as used in the Letokhov model^2,20^ and consider analytically the role of different fractional operators on the critical amplification volume of one-dimensional (1D) random media with gain.

We now introduce the single-order time-fractional generalization of the Letokhov model, which macroscopically describes sub-diffusive transport in a correlated random medium with gain. In order to generalize the standard diffusion equation to its sub-diffusion counterpart^17^, it is necessary to replace the time derivative with a fractional derivative of order *α* (0 < *α* < 1) and the classical diffusion constant D with a constant *K*_*α*_ that has units *m*^2^*s*^−*α*^. Therefore, working in scaled (adimensional) time *t*′ we can write the single-order fractional diffusion-reaction equation as:

7 $${D}_{t^{\prime} }^{\alpha }W^{\prime} (x,t^{\prime} )={K}_{\alpha }{\tau }_{d}^{\alpha }\frac{{\partial }^{2}W^{\prime} (x,t^{\prime} )}{\partial {x}^{2}}+{(\frac{{\tau }_{d}}{{\tau }_{g}})}^{\alpha }W^{\prime} (x,t^{\prime} )$$

where ${D}_{t^{\prime} }^{\alpha }$ is the Caputo fractional derivative operator in dimensionless time and:

8 $${K}_{\alpha }=\frac{{\rm{\Gamma }}(\alpha +1)\langle {x}^{2}(t)\rangle }{2{t}^{\alpha }}$$

is the expression of the generalized diffusion coefficient^17^, which reduces to the standard value *D* when *α* = 1. However, when *α* < 1 Eq. (7) macroscopically describes the anomalous regime of sub-diffusion photon transport^17^.

Similarly to the case of the classical Letokhov model we proceed to obtain the solution of the single-order fractional model using the separation of variables technique. The spatial part of the separated solution is the same as for the Letokhov case, and it will not be discussed further. Here we are interested in understanding the build-up of the energy density in the medium versus time, i.e. in the behavior of the time-dependent part of the separated solution *f*(*t*′). This can be analytically obtained via Laplace transformation, yielding^21^:

9 $$\hat{f}(s)=f(0)\frac{{s}^{\alpha -1}}{{s}^{\alpha }+{B}_{n}^{2}{K}_{\alpha }{\tau }_{d}^{\alpha }-{({\tau }_{d}/{\tau }_{g})}^{\alpha }}$$

where *s* is the Laplace transform variable conjugate to *t*′, and *B*_*n*_ is the eigenvalue of Eq. 4. The time-dependent solution *f*(*t*′) can be obtained by inverse Laplace transformation, which can be written in closed-form by using the Mittag-Leffler function *E*_*α*_^22^ as:

10 $$f(t)={E}_{\alpha }[A(\alpha ){t}^{\alpha }]=\sum _{n=0}^{{\rm{\infty }}}\,\frac{{(A(\alpha ){t}^{\alpha })}^{n}}{{\rm{\Gamma }}(\alpha n+1)}$$

where $A(\alpha )={({\tau }_{d}/{\tau }_{g})}^{\alpha }-{B}_{n}^{2}{K}_{\alpha }{\tau }_{d}^{\alpha }$ is the function that determines the corresponding lasing threshold. We can note that similarly to the classical Letokhov model, Eq. (10) predicts that, for a given value of the gain length ${\ell }_{g}$, the time-dependent solution of the fractional transport equation will increase in time when *A*(*α*) > 0 and decrease when *A*(*α*) < 0. Thus, the lasing threshold for our fractional in time generalization of the Letokhov model is provided by the condition:

11 $${({\tau }_{d}/{\tau }_{g})}^{\alpha }-{B}_{n}^{2}{K}_{\alpha }{\tau }_{d}^{\alpha }=0$$

Considering only the fundamental mode ${B}_{1}=\frac{\pi }{L}$ we can obtain a simple expression for the critical amplification length *L*_*α*_ and consequently the critical lasing volume:

12 $${L}_{\alpha }^{2}={\pi }^{2}{K}_{\alpha }{(\frac{{\ell }_{g}}{v})}^{\alpha }\Rightarrow {V}_{\alpha }={\pi }^{3}{K}_{\alpha }^{3/2}{(\frac{{\ell }_{g}}{v})}^{\frac{3\alpha }{2}}$$

The asymptotic scaling versus time *t* of the sub-diffusive equation is known to be described by the law^17^:

13 $$\langle {x}^{2}(t)\rangle =\frac{2{K}_{\alpha }{t}^{\alpha }}{{\rm{\Gamma }}(\alpha +1)}$$

Moreover, the mean-squared displacement (MSD) 〈*x*^2^(*t*)〉 and time *t* are proportional to ${\ell }_{t}^{2}$ and ${\ell }_{t}/v$, respectively, so that we can write in dimensional units the equation:

14 $$\frac{{\rm{\Gamma }}(\alpha +1)\langle {x}^{2}(t)\rangle }{2{t}^{\alpha }}=c\frac{{\ell }_{t}^{2}}{{({\ell }_{t}/v)}^{\alpha }}={K}_{\alpha },$$

where *c* is a constant. Since we require that *K*_1_ = *D*, we obtain $c=\frac{1}{2}$ and therefore we can rewrite the generalized diffusion constant as:

15 $${K}_{\alpha }=\frac{1}{2}{\ell }_{t}^{2-\alpha }{v}^{\alpha }$$

We note that Eq. (14) is also supported by the so-called aging argument, as detailed in ref.^23^. In short, this is based on the fact that sub-diffusion processes are always associated to memory effects in time. Consequently, at short times after a sub-diffusive transport process starts we can always write $t \sim {\tau }_{d}={\ell }_{t}/v$, exactly like in any traditional (memory-less) diffusion process governed by the parameters $v,{\ell }_{t}$. Therefore, in this short-time regime we have $\langle {x}_{\alpha }^{2}({\tau }_{d})\rangle  \sim \langle {x}^{2}({\tau }_{d})\rangle$. Such an initial-time condition yields ${K}_{\alpha } \sim D{(\frac{{l}_{t}}{v})}^{1-\alpha }$ which, when substituting $D=v{\ell }_{t}/2$, agrees exactly with our result in Eq. (15). Finally, we now combine Eqs (12) and (14) and obtain an analytical expression for the critical amplification volume that corresponds to a fractional derivative in time of order *α*:

16 $${V}_{\alpha }={\pi }^{3}{(\frac{{\ell }_{g}}{{\ell }_{t}})}^{\frac{3\alpha }{2}}{(\frac{{\ell }_{t}}{\sqrt{2}})}^{3}$$

Our result above shows that for a gain length ${\ell }_{g}$ larger than ${\ell }_{t}$ we have:

17 $${V}_{\alpha }\le {V}_{cri}$$

so that the critical amplification volume can be significantly smaller for a fractional random laser that operates in the sub-diffusive transport regime compared to its non-fractional (standard diffusion) counterpart. This fact motivates the engineering of correlated random scattering media in order to achieve better performances than what possible with homogeneously disordered active materials.

**Figure 1:**
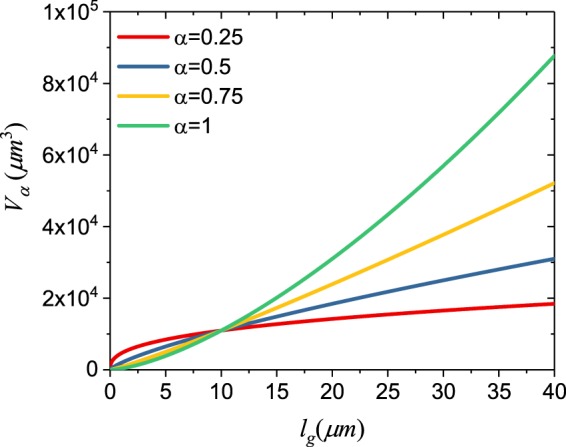
Critical volume with respect to the gain length for different sub-diffusive time fractional orders *α* and for the Letokhov model. A value of ${\ell }_{t}=10\,{\mu }m$ was used in this example.

**Figure 2:**
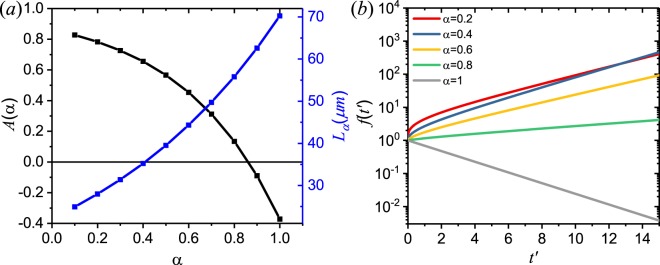
(**a**) Threshold parameter A(*α*) and critical amplification length *L*_*α*_ as a function of the time fractional order *α*, for a fixed system length L = 60 *μ*m. (**b**) Time evolution of the solution *f*(*t*) for different values of A(*α*) from panel (a).

Figure 1 shows the calculated critical length for a sub-diffusive fractional random laser with respect to the gain length for different values of the fractional order α. The case of *α* = 1 corresponds to the Letokhov model, which shows significantly larger values of *V*_*cri*_ when ${\ell }_{g} > {\ell }_{t}$, i.e., when scattering plays a role in photon amplification. We remind that small values of the fractional order *α* indicate strong photon sub-diffusion in the medium. In Fig. 2(a) we show the behavior of the critical amplification length *L*_*α*_ and of the function *A*(*α*) with respect to *α*. Interestingly, we observe that at small values *α* (i.e. for strong sub-diffusion) the reduced critical amplification length is accompanied by a larger value of *A*(*α*), which gives rise to light amplification. This behavior is also reflected in Fig. 2(b) that additionally shows the time evolution of the solution of Eq. (7) for different values of *α*. In particular, we find that by increasing the fractional order up to the threshold value *α* = 0.83, the emitted photon flux *f*(*t*′) increases less rapidly with time, and that when *α* > 0.83 (so that *A*(*α*) < 0) the solution *f*(*t*′) starts to decrease and no amplification takes place in the medium. Because we choose the system’s length *L* to be smaller than the *L*_*cri*_ of the classical Letokhov model that correspond to *α* = 1, in Fig. 2(b) the photon flux for such a model is decreasing in time. These results clearly demonstrate how, for a fixed value of the gain length ${\ell }_{g}$ of the system, fractional random lasers dramatically benefit from the slower sub-diffusive photon transport in the active medium compared to traditional random lasers.

We can quantify the benefits of the sub-diffusive transport in random lasing by defining the figure of merit *η*_*α*_ for the single-order sub-diffusive case as:

18 $${\eta }_{\alpha }={V}_{cri}/{V}_{\alpha }={(\frac{{\ell }_{g}}{{\ell }_{t}})}^{\frac{3(1-\alpha )}{2}}$$

where *V*_*cri*_ is the critical amplification volume of the integer order case (*α* = 1, standard diffusion). Calculated values of *η*_*α*_ for different choices of ${\ell }_{g}$ at three fixed values of *α* and for ${\ell }_{t}$ =  10 *μ*m are shown in Table 1. In all cases we notice that a smaller *α* value leads to a larger *η* value, with up to two orders of magnitude decrease in the critical amplification volume of fractional random lasers at the largest gain length that we considered. The table also displays the corresponding values of *η* obtained considering ultra-slow photon diffusion processes modeled by the distributed-order (DO) in time fractional transport models discussed in the next section.

**Table 1:** Figures of merit *η* for different fractional models with fixed *l*_*t*_ = 10 *μm*.

*l*_*g*_(*μm*)	*α* = 0.2	*α* = 0.5	*α* = 0.8	DO−U	DO−PL, *ν* = 2	DO−PL, *ν* = 4	DO−PL, *ν* = 50
	*η*_*α*_	*η*_*α*_	*η*_*α*_	*η*_*DO*− *U*_	*η*_*DO*− *PL*_	*η*_*DO*− *PL*_	*η*_*DO*− *PL*_
100	15.8	5.6	2.0	7.7	4.0	2.3	1.1
200	36.4	9.5	2.5	16.0	6.7	3.0	1.1
400	83.7	16.0	3.0	34.4	11.8	4.2	1.1
600	136.1	21.6	3.4	54.7	16.8	5.2	1.1
800	192.2	26.7	3.7	76.5	21.7	6.0	1.1
1000	251.2	31.6	4.0	99.7	26.6	6.8	1.2

## Fractional Random Lasers: Distributed-Order Sub-Diffusive Regime

We now further generalize the the time-fractional diffusion Eq. (7) by considering time-fractional derivative of distributed-order (DO). DO time-fractional diffusion equations have been shown to effectively describe the complex transport properties of multi-scale structures and non-homogeneous random media with ultra-slow kinetics^19,24^. Microscopically, such situations correspond to the dynamics of non-Markovian random walk processes that are asymptotically characterized by logarithmic scaling versus time of the MSD.

A DO sub-diffusion equation can be obtained when integrating with respect to a given distribution of fractional orders a single-order fractional transport equation such as the one in Eq. (7). Therefore, the prototypical DO sub-diffusion equation model is formulated as^24^:

19 $${\int }_{0}^{1}d\alpha {\tau }_{d}^{\alpha }p(\alpha ){D}_{t}^{\alpha }W=\tilde{K}{\tau }_{d}\frac{{\partial }^{2}W}{\partial {x}^{2}}$$

where $\tilde{K}$ is a constant parameter with dimensions *m*^2^*s*^−1^ and the fractional derivative operator ${D}_{t}^{\alpha }$ is averaged by the integration with a normalized distribution function (or weighting function) *p*(*α*) ≥ 0 that satisfies ${\int }_{0}^{1}\,p(\alpha )d\alpha =1$. In order to obtain a DO diffusion reaction equation consistent with the single-order sub-diffusive Eq. (7), we need to average the gain coefficient ${(\frac{{\tau }_{d}}{{\tau }_{g}})}^{\alpha }$ according to the distribution function. It is important to realize from Eq. (15) that for any 0 < *α* < 1 the quantity ${K}_{\alpha }{\tau }_{d}^{\alpha }=D{\tau }_{d}$ does not depend on the order of the fractional time derivative. Hence, it is not necessary to perform the weighted average of the coefficient in front of the second order spatial derivative. Therefore, we model a DO-fractional random laser using the diffusion-reaction equation:

20 $${\int }_{0}^{1}d\alpha p(\alpha ){D}_{{t}^{\text{'}}}^{\alpha }W\text{'}=\tilde{K}{\tau }_{d}\frac{{\partial }^{2}W}{\partial {x}^{2}}+W\text{'}(x,t\text{'})\,{\int }_{0}^{1}\,d\alpha {(\frac{{\tau }_{d}}{{\tau }_{g}})}^{\alpha }p(\alpha )$$

Notice that the equation above recovers exactly the single-order sub-diffusive Eq. (7) when *p*(*α*) = *δ*(*α* − *α*′) and $\tilde{K}=D$. The meaning of the coefficient $\tilde{K}$ is discussed in more detail in the methods section.

**Figure 3:**
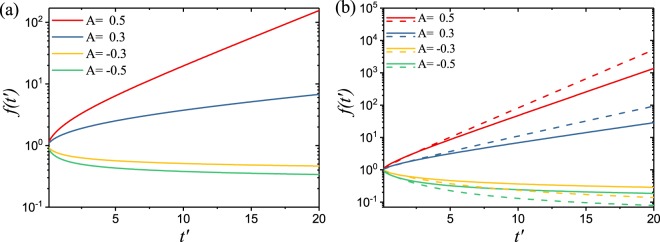
(**a**) Time evolution of the solution *f*(*t*′) of the DO−U fractional laser as a function of the threshold function A. (**b**) Time evolution of the solution *f*(*t*′) of the DO−PL fractional laser with *ν* = 2 (continuous lines) and *ν* = 4 (dashed lines) as functions of the threshold function A.

By using the separation of variables technique and the Laplace transform method^25^, the time-dependent part of the solution to the DO Eq. (20) can be readily obtained in the Laplace domain:

21 $$\hat{f}(s)=f(0)\frac{I(s)/(s)}{I(s)+\tilde{K}{\tau }_{d}{B}_{1}^{2}-I(s=\frac{{\tau }_{d}}{{\tau }_{g}})}=f(0)\frac{I(s)/(s)}{I(s)-A}$$

where *B*_1_ is the lowest eigenvalue from Eq. (4), $I(s)={\int }_{0}^{1}p(\alpha ){s}^{\alpha }d\alpha$, and $A=I(s=\frac{{\tau }_{d}}{{\tau }_{g}})-\tilde{K}{\tau }_{d}{B}_{1}^{2}$ is the threshold function for the DO case. In Fig. 3, we evaluate numerically the inverse Laplace transform of Eq. (21) for two different distribution functions: the DO uniform case (DO−U) where *p*(*α*) = 1 and the power-law distribution function (DO−PL) that corresponds to $p(\alpha )=v{\alpha }^{v-1}$. In particular, when $v=4$ this model produces logarithmic in time Sinai-type sub-diffusion^19^. We observe from Fig. 3 that *f*(*t*′) is increasing with time when *A* is positive and it is decreasing when *A* is negative, consistently with the lasing threshold condition *A* = 0. Moreover for the DO−U case we have $I(s)=\frac{s-1}{\mathrm{log}(s)}$ so that we can obtain the following expression for the critical amplification length *L*_*DO*−*U*_ from the condition:

22 $$I(s=\frac{{\tau }_{d}}{{\tau }_{g}})-\frac{{\pi }^{2}{\ell }_{t}^{2}}{2{L}_{DO-U}^{2}}=\frac{1-{\ell }_{t}/{\ell }_{g}}{\mathrm{log}\,({\ell }_{g}/{\ell }_{t})}-\frac{{\pi }^{2}{l}_{t}^{2}}{2{L}_{DO-U}^{2}}=0.$$

where we used again the expression for the lowest eigenvalue *B*_1_ = *π*/*L*. Finally we obtain the critical amplification length as:

23 $${L}_{DO-U}=\pi {\ell }_{t}\sqrt{\frac{1}{2}\frac{{\rm{l}}{\rm{o}}{\rm{g}}\,({\ell }_{g}/{\ell }_{t})}{(1-{\ell }_{t}/{\ell }_{g})}}$$

and the corresponding critical volume *V*_*DO*−*U*_. Recalling that the critical volume for the Letkhov model is ${V}_{cri}={\pi }^{3}{({\ell }_{g}{\ell }_{t}/2)}^{\frac{3}{2}}$, we can derive the figure of merit for the DO−U case as:

24 $${\eta }_{DO-U}=\frac{{V}_{cri}}{{V}_{DO-U}}={(\frac{{\ell }_{g}/{\ell }_{t}-1}{{\rm{l}}{\rm{o}}{\rm{g}}({\ell }_{g}/{\ell }_{t})})}^{\frac{3}{2}}$$

Representative values of this figure of merit are shown for different values of the amplification length in Table 1. An alternative derivation of this figure of merit based on the scaling of the MSD in DO-fractional equations is reported in the methods section.

We now consider the solution of a DO fractional laser with power-law distribution function *p*(*α*) = *να*^*ν*−1^, referred to as the DO−PL case. The DO−U is clearly a special case of DO−PL corresponding to *ν* = 1.

Figure 3(b) shows the monotonic trend of *f*(*t*′) determined by the sign of the threshold function *A* plotted for two different values of *ν*. Therefore, we can follow the same solution method based on the Eq. (21) and obtain the critical amplification length for the DO−PL fractional laser in the form:

25 $${L}_{DO-PL}=\pi {\ell }_{t}\sqrt{\frac{1}{2I(s=\frac{{\tau }_{d}}{{\tau }_{g}})}}$$

Therefore, we can write the figure of merit of the DO−PL random laser as:

26 $${\eta }_{DO-PL}=\frac{{V}_{cri}}{{V}_{DO-PL}}={[\frac{{\ell }_{g}}{{\ell }_{t}}I(s=\frac{{\tau }_{d}}{{\tau }_{d}})]}^{\frac{3}{2}}$$

However, the expression above involves the integral $I(s)={\int }_{0}^{1}\,{s}^{\alpha }\nu {\alpha }^{\nu -1}d\alpha$ that cannot be evaluated analytically. Therefore we evaluated it numerically as explained in detail in the methods section. Finally in Table 1 we show the computed values of the figure of merit for the DO−PL case at different values of ${\ell }_{g}$ and for three different values of the exponent *ν*.

**Figure 4:**
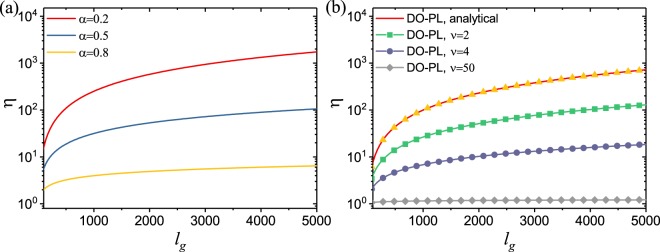
(**a**) The figure of merit *η* obtained analytically as a function of ${\ell }_{g}$ for the time fractional single-order diffusion-reaction equation and (**b**) the numerical solutions obtained for the DO−U and DO−PL fractional processes with different *ν* values. The continuous red line is the analytical solution of the DO−U case.

To better appreciate the behavior of the figure of merit beyond what reported in Table 1, we computed the analytical and the numerical solutions for *η* over an extended range of ${\ell }_{g}$ for a fixed ${\ell }_{t}=10\mu m$. Our results are shown in Fig. 4(a,b) on a semi-logarithmic scale where we compare the behavior of *η* for the cases of fractional single-order, DO−U, and DO−PL random lasers. The data in Fig. 4(a) clearly demonstrate how disordered gain media with strong sub-diffusive behavior, which are described by smaller values of the fractional order *α*, feature the largest value of *η*. In Fig. 4(b) we show our results for the DO fractional lasers with different distribution functions, which are obtained numerically from Eq. (25).

Our findings demonstrate that the figure of merit *η* for the fractional random lasers is maximized for the DO sub-diffusion process (when ${\ell }_{g}\gg {\ell }_{t}$) with a uniform distribution function (i.e. DO−U), which is a special case of the power-law distribution function (DO−PL) obtained for *ν* = 1. This is to be expected on the basis that DO−U sub-diffusion describes transport in the most heterogeneous environments where the multi-scale structural complexity of the medium significantly slows down the transport dynamics at each scale promoting optical amplification.

We can directly compare the figures of merit of the fractional single-order laser and the DO−U fractional laser by considering the ratio *η*_*DO−U*_/*η*_*α*_,which can be written as:

27 $$\frac{{\eta }_{DO-U}}{{\eta }_{\alpha }}={[\frac{({\ell }_{g}/{\ell }_{t})-1}{\mathrm{log}({\ell }_{g}/{\ell }_{t}){({\ell }_{g}/{\ell }_{t})}^{1-\alpha }}]}^{\frac{3}{2}}\to \mathop{\mathrm{lim}}\limits_{{\ell }_{g}/{\ell }_{t}\to \infty }\frac{{\eta }_{DO-U}}{{\eta }_{\alpha }}={[\frac{{({\ell }_{g}/{\ell }_{t})}^{\alpha }}{\mathrm{log}({\ell }_{g}/{\ell }_{t})}]}^{\frac{3}{2}}$$

It can be noticed that the expression above exceeds unity in the limit ${\ell }_{g}/{\ell }_{t}\to \infty$ as a function of ${\ell }_{g}/{\ell }_{t}$, irrespective of the value of *α*. Therefore, random laser structures governed by ultra-slow DO diffusion processes will always outperform their single-order fractional counterparts. This conclusion is natural from a physical viewpoint since DO-fractional diffusion processes are microscopically equivalent to correlated random walks characterized at large times by a logarithmic MSD scaling, which grows much slower than any power-law achieved by single-order processes. Therefore the photon dwell time in a DO sub-diffusive gain medium is much longer than in a single-order sub-diffusive one, thus increasing the probability for amplification and reducing the critical volume. The analytical theory of the DO−U process also allows us to validate our numerical solutions for the DO−PL cases. This is done by comparing the numerical and the analytical results of the DO−U and DO−PL cases for *ν* = 1. The two curves perfectly overlap in Fig. 4(b). Furthermore, we observe that when *ν* → ∞ the distribution function of the DO−PL process converges to the Letokhov model of classical diffusion. This is also reflected by our numerical results for the DO−PL case at large *ν* values. In fact, as shown in Fig. 4(b), the value of *η*_*DO*−*PL*_ is close to unity already when *ν* = 50. A more detailed discussion on the fundamental solutions of the DO−PL model is reported in the methods section.

## Fractional Random Lasers: Super-Diffusive Regime

We now discuss the role of photon super-diffusion in the context of fractional random lasers. Super-diffusion processes are elegantly described within the framework of fractional calculus by considering spatial derivatives of fractional order^17^. Super-diffusion of light has been experimentally demonstrated in recent years in relation to Lévy flights^26,27^ which are scale-invariant correlated random walks with a step length described by a probability distribution with heavy tails, implying diverging moment^28,29^.

Therefore, we now consider the reaction-diffusion space-fractional equation:

28 $$\frac{\partial W(x,t)}{\partial t}={K}_{\beta }{D}^{\beta }W(x,t)+\frac{v}{{\ell }_{g}}W(x,t)$$

where the *D*^*β*^ is the Caputo derivative in space (1 < *β* < 2) and *K*_*β*_ is the diffusion coefficient that measures the asymptotic (long-time) limit of the ratio between the fractional spatial moment $\langle |x{|}^{\beta }\rangle$ and time^17^. Note that in the case of photon super-diffusion we do not need to scale the gain coefficient because the fractional process only affects the spatial operators. Using again the separation of variables technique it is straightforward to obtain the time evolution part of the solution in the form:

29 $$f(t)=f(0){e}^{{(\frac{v}{{\ell }_{g}}-{K}_{\beta }{C}^{\beta })}^{t}}$$

where *C* is a constant that will be determined below. The expression above is similar to the case of single-order sub-diffusion in time and the laser action (i.e. growing photon flux with time) will start when the following condition is met:

30 $$C={(\frac{v}{{\ell }_{g}{K}_{\beta }})}^{\frac{1}{\beta }}$$

The value of the constant *C* can be found by solving the differential equation for the spatial part of the solution:

31 $${D}^{\beta }\varphi (x)+{C}^{\beta }\varphi (x)=0$$

For super-diffusive transport the spatial dependent solution *ϕ*(*x*) can be explicitly obtained as^18^:

32 $$\varphi (x)={c}_{1}{E}_{\beta }(-{(Cx)}^{\beta })+{c}_{2}{J}^{1}[{E}_{\beta }(-{(Cx)}^{\beta })]$$

where *E*_*β*_ denotes the Mittag-Leffler function and ${J}^{1}{E}_{\beta }(\,-\,{(Cx)}^{\beta })={\int }_{0}^{x}\,dx\text{'}{E}_{\beta }(\,-\,{(Cx\text{'})}^{\beta })$. The constants *C* and the ratio $\frac{{c}_{1}}{{c}_{2}}$ are determined by imposing the zero boundary conditions to *ϕ*(*x*) at *x* = 0 and *x* = *L* as discussed in detail in ref.^24^. For the two terms in Eq. (32) at x = 0 we have ${J}^{1}[{E}_{\beta }(0)]={\int }_{0}^{0}\,{E}_{\beta }(0)=0$, ${E}_{\beta }(0)\ne 0$. Implementing the boundary condition *ϕ*(0) = 0, we obtain *c*_1_ = 0. When imposing the boundary condition for *ϕ*(*x*) at *x* = *L* we have *J*^1^[*E*_*β*_(−(*CL*)^*β*^)] = 0. If we define *λ* = *Cx* and recall Eqs (9) and (10) we obtain:

33 $$ {\mathcal L} [{J}^{1}[{E}_{\beta }(-{(\lambda )}^{\beta })]](s)=\frac{{s}^{\beta -2}}{{s}^{\beta }+1}.$$

The equation above can be Laplace inverted numerically to find the zeros λ^*^ of the function *J*^1^[*E*_*β*_(−(*λ*)^*β*^)] for *λ* > 0. We report in Table 2 the values of the zeros λ^*^ computed for different values of *β*.

**Table 2:** Zero positions λ^*^ of *J*^1^[*E*_*β*_(−(*λ*)^*β*^)].

	*β* = 1.95	*β* = 1.90	*β* = 1.75	*β* = 1.60
*λ* ^*^	3.20	3.27	3.64	5.35

We note that for 1 < *β* < 1.6, no zeros can be found when *λ* > 0, except for trivial (i.e. identically zero) solutions. Finally, from the boundary condition *ϕ*(*L*) = 0 we obtain $C\approx \frac{1}{L}$ when 1.6 < *β* < 2, which yields using Eq. (30) an analytical expression for the critical lasing volume:

34 $${V}_{\beta }={L}^{3}\approx {(\frac{{K}_{\beta }}{v}{\ell }_{g})}^{\frac{3}{\beta }}$$

Spatial super-diffusion arises in the context of continuous time random walk (CTRW) processes where the jump length and the waiting time probability density functions (pdf) follow power-law distributions^17^. In this context the root mean squared displacement (rms fractional moment) of the solution is proportional to the minimal jump length, which is ${\ell }_{t}$. Remembering that time is expressed in unites of $\frac{{\ell }_{t}}{v}$, we see that ${K}_{\beta } \sim {\ell }_{t}^{\beta -1}v$ from which we can estimate the critical lasing volume as:

35 $${V}_{\beta }\propto {\ell }_{t}^{3}{(\frac{{\ell }_{g}}{{\ell }_{t}})}^{\frac{3}{\beta }}$$

Despite we cannot find an analytical expression for this critical volume, the expression above makes us fully appreciate that when ${\ell }_{g}\gg {\ell }_{t}$ the super-diffusive regime will not result in any advantage in lasing amplification volume compared to the sub-diffusive regimes previously discussed. In fact, for the super-diffusive case our scaling argument shows that *V*_*β*_ > *V*_*cri*_. In addition, if we compute the figure of merit *η*_*SD*_ = *V*_*cri*_/*V*_*β*_ we have:

36 $${\eta }_{\beta }\propto {(\frac{{\ell }_{g}}{{\ell }_{t}})}^{\frac{3}{2}-\frac{3}{\beta }}$$

Since 1.6 < *β* < 2, then we have *η*_*β*_ → 0 when ${\ell }_{g}/{\ell }_{t}\to \infty$, rendering the super-diffusive fractional laser less efficient than the standard diffusion case. This is consistent with the fact that the photon dwell time in the super-diffusive medium is much smaller than in a sub-diffusive active medium, which reduces the probability for amplification resulting in larger critical volumes. A detailed discussion of the fundamental solution of the photon super-diffusive equation is provided in the methods section.

## Conclusion

Our work generalizes the concept of a random laser to fractional order operators in time and space and addresses it analytically, within the powerful framework of fractional reaction-diffusion equations, the threshold behavior of fractional random lasers. We provide a figure of merit *η* that quantifies the benefits of fractional order lasing compared to the classical Letokhov model for standard diffusion in a gain medium. Our analysis shows that random lasers that leverage engineered sub-diffusion phenomena and ultra-slow photon logarithmic in time photon transport feature significantly reduced amplification length compared to the classical Letokhov model. We also derived simple analytical relations that precisely quantify the critical lasing volumes achievable for different sub-diffusion regimes. In particular, we reveal that DO fractional lasers with uniform distribution (DO−U) are the most efficient systems among the class of DO fractional lasers and that the value of *η* is larger than the one possible with any single-order fractional laser case when ${\ell }_{g}/{\ell }_{t}\to \infty$. We finally considered the transport properties in the super-diffusive regime and demonstrated quantitatively that the corresponding figure of merit decreases when the gain length is increased, rendering super-diffusive fractional lasers less efficient and appealing compared to the ones based on both classical diffusion and sub-diffusion. Our work demonstrates the importance of engineering photon sub-diffusion phenomena in active photonic devices and can pave the way to the realization of novel random lasers and aperiodic structures with designed correlated disorder and anomalous transport properties resulting in dramatically reduced amplification threshold and footprint. Finally, we remark that since photon sub-diffusive phenomena in non-resonant fractional lasers do not rely on wave interference effects, such mechanisms are intrinsically broadband and can open fascinating scenarios for the engineering of active photonic devices beyond random lasers, such as more efficient optical sensors, nonlinear optical converters, and energy harvesting devices. We believe that our study will stimulate future work on the engineering of fractional photon transport in random lasers that can be experimentally realized using correlated disordered media, metamaterials, or deterministic aperiodic structures.

## Methods

### Fundamental solutions of different models

**Figure 5:**
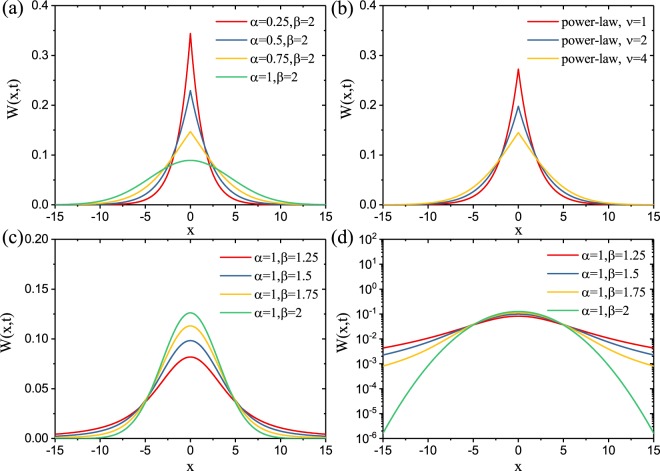
(**a**) The fundamental solution *W*(*x*, *t* = 10) of single-order sub-diffusion for different fractional time derivative order *α*. (**b**) The fundamental solution *W*(*x*, *t* = 10) of DO diffusion follows power-law distribution function for *ν* = 1(DO−U), *ν* = 2, and *ν* = 4. (**c**) The fundamental solution *W*(*x*, *t* = 10) of super-diffusion for different fractional spatial derivative *β* in linear scale. (**d**) The fundamental solution *W*(*x*, *t* = 10) of super-diffusion as shown in (**c**) in the logarithmic scale.

In this section we provide more detailed information on the analytical and numerical methods utilized to obtain the main results presented in the text. In particular, we will focus on the rigorous derivations of the fundamental solutions of different sub-diffusive and super-diffusive transport models. We plot in Fig. 5 the fundamental solutions obtained for sub-diffusive, distributed-order (DO−U and DO−PL), and super-diffusive transport. All solutions have been obtained considering a generalized diffusion coefficient equal to unity.

Fundamental solutions are obtained by solving the initial-value Cauchy problem for different fractional diffusion equations. The single-order sub-diffusion Cauchy problem is defined by^25^:

37 $$\{\begin{array}{rcl}{D}^{\alpha }W(x,t) & = & {K}_{\alpha }\frac{{\partial }^{2}W(x,t)}{\partial {x}^{2}}\\ W(x,0) & = & \delta (x)\end{array}$$

The equation solution is discussed in detail in ref.^25^. The Laplace domain expression of the fundamental solution $\tilde{W}(x,s)$, where *s* is the Laplace domain argument, can be written as:

38 $$\tilde{W}(x,s)=\frac{1}{2\sqrt{{K}_{\alpha }}}{s}^{\frac{\alpha }{2}-1}\,\exp (-\frac{|x|}{\sqrt{{K}_{\alpha }}}{s}^{\frac{\alpha }{2}})$$

Following the approach in ref.^30^ we perform a numerical inverse Laplace transform and plot in Fig. 5(a) the single-order sub-diffusion fundamental solutions *W*(*x*, *t*) for different *α* at time *t* = 10 and for a generalized diffusion coefficient *K*_*α*_ = 1. We can observe that for *α* = 1 the result recovers to the well-known Gaussian fundamental solution^25^. However, when decreasing *α*, the *W*(*x*, *t*) solution increases in amplitude at the center where it develops a characteristic kink and shows small tails for large values of the space coordinate *x*. This shows that a smaller order *α* of the generalized time derivative leads to a slower diffusion process. The fundamental solution of the DO-fractional diffusion is obtained by solving the equation^25^:

39 $$\{\begin{array}{rcl}{\int }_{0}^{1}p(\alpha )[{D}^{\alpha }W(x,t)]d\alpha  & = & \tilde{K}\frac{{\partial }^{2}W(x,t)}{\partial {x}^{2}}d\alpha \\ W(x,0) & = & \delta (x)\end{array}$$

The fundamental solution *W*(*x*, *t*) in the Laplace domain is expressed as^25^:

40 $$\tilde{W}(x,s)=\frac{I{(s)}^{\frac{1}{2}}}{2s\sqrt{\tilde{K}}\exp \,[-|x|{(I(s)/\sqrt{\tilde{K}})}^{\frac{1}{2}}]}$$

In Fig. 5(b) we show the numerically obtained fundamental solution for the DO−U model at *t* = 10. For the DO−PL case, the expression of *I*(*s*) is:

41 $$I(s)={\int }_{0}^{1}\,{s}^{\alpha }\nu {\alpha }^{\nu -1}d\alpha =\nu [\gamma (\nu ,\,-\,\mathrm{log}(s))]{(-\mathrm{log}(s))}^{-\nu }$$

where the *γ*(*ν*, −log(*s*)) is the lower incomplete gamma function defined as $\gamma (s,x)={\int }_{0}^{x}\,{t}^{s-1}{e}^{-t}dt$. Implementing directly the inverse Laplace transform on Eq. (41) is difficult because *s* is contained in the lower limit of the integral that defines *γ*. Therefore, we expanded this integral using the holomorphic extension of *γ*(*ν*, −log(*s*)) as following^31^:

42 $$\gamma (\nu ,\,-\,\mathrm{log}(s))={[-\mathrm{log}(s)]}^{\nu }{\rm{\Gamma }}(\nu )s\sum _{k=0}^{\infty }\,\frac{{(-\mathrm{log}(s))}^{k}}{{\rm{\Gamma }}(\nu +k+1)}$$

Combining Eqs (40–42), we can now perform the numerical inverse Laplace transform and obtain the fundamental solution for different DO−PL cases. We plot in the Fig. 5(b) the fundamental solutions of DO−PL with *ν* = 2 and *ν* = 4 at *t* = 10. We can observe that by increasing *ν* from 1 to 4, the fundamental solution is decreasing in its value at the center and spreads spatially with longer tails indicating a faster diffusion. Therefore, the slower diffusion for the DO−PL case with *ν* = 1, which coincides with the DO−U model, leads to larger *η* values as shown in Table 1.

**Figure 6:**
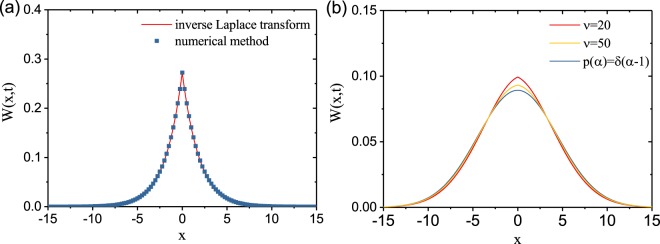
(**a**) The fundamental solution *W*(*x*, *t*) from inverse Laplace transform and our numerical method for DO−PL, *ν* = 1 when *t* = 10. (**b**) The fundamental solution *W*(*x*, *t*) of standard diffusion and DO−PL obtained from our numerical method for *ν* = 20 and *ν* = 50 when *t* = 10.

Below we provide an additional validation of the numerical method we used for obtaining the DO−PL fundamental solutions. In Fig. 6(a), we show the results obtained from the inverse Laplace transform of Eq. (40) when substituting $I(s)=\frac{s-1}{\mathrm{log}(s)}$ and by using the holomorphic extension method for DO−PL with *ν* = 1. The results obtained using the two methods overlap completely, as shown in 6(a), fully validating our numerical approach. Moreover, the DO−PL recovers the standard diffusion solution when *α* → ∞. We plot the results of DO−PL with *ν* = 20 and *ν* = 50 in Fig. 6(b). Our numerical results approach the expected Gaussian line shape when increasing the values of *ν*. Interestingly there is an anlytical expression for the fundamental solution of the DO−PL model for *t* ≫ 1, which is given by^32^:

43 $$W(x,t)\approx \frac{1}{\sqrt{4\tilde{K}}}{[\frac{{\rm{\Gamma }}(\nu +1)}{\mathrm{log}{(t)}^{\nu }}]}^{1/2}\exp \{-{(\frac{{\rm{\Gamma }}(\nu +1)}{\tilde{K}})}^{1/2}\frac{|x|}{\mathrm{log}\,{(t)}^{\nu /2}}\}$$

We find an excellent agreement between our numerical results obtained with the holomorphic extension method and the asymptotic analytical expression above when *t* > 500, whcih further validates our approach.

Finally, the fundamental solution for the super-diffusive case can be obtained by solving the equation:

44 $$\{\begin{array}{rcl}\frac{\partial W(x,t)}{\partial t} & = & {K}_{\beta }{D}^{\beta }W(x,t)\\ W(x,0) & = & \delta (x)\end{array}$$

Using Fourier transformation in space we can derive the fundamental solution *W*(*k*, *t*) in the form^17^:

45 $$\tilde{W}(k,t)=\exp (\,-\,{K}_{\beta }t|k|)$$

where *k* is the Fourier domain argument. In Fig. 5(c,d) we show the corresponding fundamental solutions *W*(*x*, *t*) at *t* = 10 obtained by performing numerical Fourier inversion. We can observe that by decreasing *β* the fundamental solution will develop longer (heavy) tails in the spatial domain, which imply the divergence of its finite moments. This is more evident in Fig. 5(d) where we plot the solutions using semi-logarithmic scale. In Fig. 5(d) we also plot in green the fundamental solution of the standard diffusion model (*α* = 1, *β* = 2), which clearly shows slower diffusion fronts compared to the super-diffusive cases. This behavior explains why super-diffusive fractional lasers will not benefit from reduced critical amplification volumes when compared to even the traditional Letokhov model.

### Diffusion constant in the uniformly distributed- order case

In our evaluation of *V*_*DO*−*U*_ and *V*_*DO*−*PL*_ we used the $\tilde{K}=D$, where *D* is the classical diffusion constant. This assumption is justified by the aging phenomena, as explained in details in ref.^23^. The time sub-diffusion reflects long-term memory effects. Consequently in the first steps of the process, when *t* ~ *τ*_*d*_ = *l*_*t*_/*v*, it behaves like a classical diffusion with the same parameters *v*, *l*_*t*_. Even if asymptotically classical diffusion and DO−U are totally different processes, we can expect that they give the same result at the beginning. Therefore, using the expression of $\langle x{(\tau )}^{2}\rangle$ for DO−U and strandard diffusion we obtain:

46 $$ < x{(\tau )}^{2}{ > }_{DO-U}=2\tilde{K}\tau (\gamma +e\ast {E}_{1}(1))\approx 2\tilde{K}\tau \ast 1.17\approx  < x{(\tau )}^{2}{ > }_{cla}=2D\tau $$

where *γ* ≈ 0.577 is the Euler-Mascheroni constant and ${E}_{1}(z)={\int }_{z}^{\infty }\,dy\frac{{e}^{-y}}{y}$ is the exponential integral. Eq. (46) confirms that *D* is a good approximation for $\tilde{K}$, as we used in the main text.

### Scaling argument for the threshold condition of DO fractional lasers

A simple derivation based on MSD scaling provides an estimate of the lasing threshold for DO fractional lasers with uniform and power-law distribution. Let us consider a photon diffusing (classically or anomalously) in a active medium. A photon is amplified at least on average once before leaving the sample at the lasing threshold condition. With initial condition *W*(*x*,0) = *δ*(*x*), the expression of $\langle x{(\tau )}^{2}\rangle$ for DO−U can be found^24^ as follows:

47 $$\langle x{(t)}^{2}\rangle =2\tilde{K}{\tau }_{d}[\mathrm{log}\,(\frac{t}{{\tau }_{d}})+\gamma +{e}^{\frac{t}{{\tau }_{d}}}{E}_{1}(\frac{t}{{\tau }_{d}})]$$

Assuming that the diffusion process is the same in both directions $\overrightarrow{x}$ and $-\overrightarrow{x}$, on average a photon can travel a root-mean-squared (rms) distance equal to *L*/2 before leaving the sample. Therefore, we consider ${L}_{cri}/2\approx {\ell }_{amp}$, where ${\ell }_{amp}$ is the rms distance corresponding to the time for propagation over a length ${\ell }_{g}$. Therefore we can write:

48 $${\ell }_{amp}^{2}=\langle x{(\frac{{\ell }_{g}}{v})}^{2}\rangle ={\ell }_{t}^{2}[\mathrm{log}\,(\frac{{\ell }_{g}}{{\ell }_{t}})+\gamma +{e}^{\frac{{\ell }_{g}}{{\ell }_{t}}}{E}_{1}(\frac{{\ell }_{g}}{{\ell }_{t}})]$$

The critical amplification volume *V*_*DO*−*U*_ for the DO−U random laser follows as:

49 $${V}_{DO-U}={(2{\ell }_{amp})}^{3}={(2{\ell }_{t})}^{3}{[\mathrm{log}(\frac{{\ell }_{g}}{{\ell }_{t}})+\gamma +{e}^{\frac{{\ell }_{g}}{{\ell }_{t}}}{E}_{1}(\frac{{\ell }_{g}}{{\ell }_{t}})]}^{\frac{3}{2}}$$

The estimated figure of merit ${\eta ^{\prime} }_{DO-U}$ can now be explicitly obtained as:

50 $${\eta ^{\prime} }_{DO-U}={V}_{cri}/{V}_{DO-U}={(\frac{\pi }{2})}^{3}{(\frac{{\ell }_{g}}{2{\ell }_{t}})}^{\frac{3}{2}}{\textstyle /}{[{\rm{l}}{\rm{o}}{\rm{g}}(\frac{{\ell }_{g}}{{\ell }_{t}})+\gamma +{e}^{\frac{{\ell }_{g}}{{\ell }_{t}}}{E}_{1}(\frac{{\ell }_{g}}{{\ell }_{t}})]}^{\frac{3}{2}}$$

When computed for the same values of the amplification length in Table 1 this simple method agrees quite well with the more rigorous derivation presented in the main text. In fact we obtain ${\eta }_{DO-U}/{\eta }_{DO-U}^{\text{'}}=0.89\pm 0.02$. The origin of this discrepancy relies on the fact that the $\langle {x}^{2}\rangle$ expression is obtained under the initial condition *W*(*x*, 0) = *δ*(*x*) and with no physical boundaries (i.e. ignoring the finite size *L* of the system). Moreover, for the rigorous result presented in Eq. (24) the figure of merit *η*_*DO*−*U*_ consistently goes to 1 when ${\ell }_{g}\to {\ell }_{t}$ as for the Letokhov model case while the estimated value ${\eta ^{\prime} }_{DO-U}$ does not.

This approximate method can however be applied to the DO−PL case. The asymptotic expression of $\langle {x}^{2}(t)\rangle$ for a DO fractional transport model with power-law distribution can be expressed as^32^:

51 $$\langle x{(t)}^{2}\rangle =\{\begin{array}{ll}\frac{2\tilde{K}}{\nu }t\,\mathrm{log}\,(\frac{\tau }{t}), & t\ll \tau \\ \frac{2\tilde{K}\tau }{{\rm{\Gamma }}(\nu +1)}\,\mathrm{log}\,{(\frac{t}{\tau })}^{\nu }, & t\gg \tau \end{array}$$

Following the same steps as for the derivation of the DO−U we can obtain the critical volume. Since in our paper we are using the condition ${\ell }_{g}\gg {\ell }_{t}$ as in ref.^20^, we should consider the second equation in (51). Therefore for the DO−PL case we can write directly:

52 $${\ell }_{amp}^{2}=\langle x{(\frac{{\ell }_{g}}{v})}^{2}\rangle =\frac{{\ell }_{t}^{2}}{{\rm{\Gamma }}(\nu +1)}{[\mathrm{log}(\frac{{\ell }_{g}}{{\ell }_{t}})]}^{\nu }$$

The critical amplification volume *V*_*DO*−*PL*_ for the DO−PL random laser follows:

53 $${V}_{DO-PL}={(2{\ell }_{amp})}^{3}=\frac{8{\ell }_{t}^{3}}{{\rm{\Gamma }}{(\nu +1)}^{\frac{3}{2}}}{[\mathrm{log}(\frac{{\ell }_{g}}{{\ell }_{t}})]}^{\frac{3\nu }{2}}$$

Finally, the figure of merit for the DO−PL model can be approximated as:

54 $${\eta ^{\prime} }_{DO-PL}={V}_{cri}/{V}_{DO-PL}={(\frac{\pi }{2})}^{3}{(\frac{{\rm{\Gamma }}(\nu +1){\ell }_{g}}{2{\ell }_{t}})}^{\frac{3}{2}}{\boldsymbol{/}}{[{\rm{l}}{\rm{o}}{\rm{g}}(\frac{{\ell }_{g}}{{\ell }_{t}})]}^{\frac{3\nu }{2}}$$

We note that the values of the ${\eta ^{\prime} }_{DO-PL}$ obtained with the simpler method describe above are found to be within 15% of the more accurate values of *η*_*DO*−*PL*_ (derived as explained in the main text) shown in Table 1 under the asymptotic condition ${\ell }_{g}\gg {\ell }_{t}$.
